# Long-term clinical course of anti-glycyl tRNA synthetase (anti-EJ) antibody-related interstitial lung disease pathologically proven by surgical lung biopsy

**DOI:** 10.1186/s12890-016-0325-y

**Published:** 2016-12-01

**Authors:** Hajime Sasano, Eri Hagiwara, Hideya Kitamura, Yasunori Enomoto, Norikazu Matsuo, Tomohisa Baba, Shinichiro Iso, Koji Okudela, Tae Iwasawa, Shinji Sato, Yasuo Suzuki, Tamiko Takemura, Takashi Ogura

**Affiliations:** 1 Department of Respiratory Medicine, Kanagawa Cardiovascular and Respiratory Center, 6-16-1 Tomioka-higashi, Kanazawa-Ku, Yokohama, 236-0051 Japan; 2 Department of Radiology, Yokohama Rosai Hospital for Labor Welfare Corporation, 3211 Kozukue-Chō, Kōhoku-Ku, Yokohama, 222-0036 Japan; 3 Department of Pathology, Yokohama City University Graduate School of Medicine, 3-9, Fukuura, Kanazawa-Ku, Yokohama, 236-0004 Japan; 4 Department of Radiology, Kanagawa Cardiovascular and Respiratory Center, 6-16-1 Tomioka-higashi, Kanazawa-Ku, Yokohama, 236-0051 Japan; 5 Department of Rheumatology, Tokai University School of Medicine, 143 Shimokasuya, Isehara, 259-1193 Japan; 6 Department of Pathology, Japan Red Cross Medical Center, 4-1-22 Hiroo, Shibuya-Ku, Tokyo, 150-8935 Japan; 7 Present Address: Department of Respiratory Medicine, Ise Red Cross Hospital, 1-471-2 Funae, Ise, 516-8512 Japan; 8 Present Address: Second Division, Department of Internal Medicine, Hamamatsu University School of Medicine, 1-20-1 Handayama, Higashi-Ku, Hamamatsu, 431-3192 Japan; 9 Present Address: Department of Respirology, Kurume University School of Medicine, 67 Asahi-Chō, Kurume, 830-0011 Japan

**Keywords:** Interstitial pneumonia with autoimmune featured (IPAF), Anti-synthetase syndrome, Dermatomyositis, Polymyositis, Idiopathic interstitial pneumonia

## Abstract

**Background:**

Anti-glycyl-tRNA synthetase (anti-EJ) antibody is occasionally positive in patients with interstitial lung disease (ILD). We aimed to define the clinical, radiological and pathological features of patients with anti-EJ antibody-positive ILD (EJ-ILD).

**Methods:**

We retrospectively analyzed the medical records of 12 consecutive patients with EJ-ILD who underwent surgical lung biopsy.

**Results:**

The median follow-up time was 74 months (range, 17–115 months). The median age was 62 years (range, 47–75 years). Seven of 12 patients were female. Eight patients presented with acute onset. Six patients eventually developed polymyositis/dermatomyositis. On high-resolution computed tomography, consolidation and volume loss were predominantly observed in the middle or lower lung zone. Nine patients presented pathologically nonspecific interstitial pneumonia with organizing pneumonia, alveolar epithelial injury and prominent interstitial cellular infiltrations whereas the other three patients were diagnosed with unclassifiable interstitial pneumonia. Although all patients but one improved with the initial immunosuppressive therapy, five patients relapsed. When ILD relapsed, four of the five patients were treated with corticosteroid monotherapy. Four of the six patients without relapse have been continuously treated with combination therapy of corticosteroid and immunosuppressant.

**Conclusions:**

Patients with EJ-ILD often had acute onset of ILD with lower lung-predominant shadows and pathologically nonspecific interstitial pneumonia or unclassifiable interstitial pneumonia with acute inflammatory findings. Although the disease responded well to the initial treatment, relapse was frequent. Because of the diversity of the clinical courses, combination therapy of corticosteroid and immunosuppressant should be on the list of options to prevent relapse of EJ-ILD.

## Background

Interstitial lung disease (ILD) is caused by various etiologies including collagen vascular disease, drug induced and inhalation exposure. Patients with ILD often have collagen vascular disease-related autoantibodies even when they do not fulfill the diagnostic criteria for any collagen vascular diseases [1]. In 2015, the “European Respiratory Society (ERS)/American Thoracic Society (ATS) Task Force on Undifferentiated Forms of Connective Tissue Disease-associated Interstitial Lung Disease” proposes the term “interstitial pneumonia with autoimmune features” (IPAF) and offers classification criteria organized around the presence of a combination of features from three domains: a clinical domain, a serologic domain and a morphologic domain [2]. Recent report showed autoantibodies against aminoacyl-tRNA synthetase (ARS) have been found to be highly specific for myositis and to associate with complicating ILD, arthritis, Raynaud’s phenomenon, mechanic’s hand and fever. Ten anti-ARS antibodies have been identified: anti-Jo-1, EJ, PL-7, PL-12, OJ, KS, Zo, SC, JS and YRS antibodies [3, 4].

Anti-ARS antibodies are positive in 20%–35% of patients with polymyositis/dermatomyositis (PM/DM) [3, 5]. Among patients with PM/DM, anti-Jo-1 antibody is the most common (15%–30%) while others are seen in less than 10% [6]. Although, anti-EJ antibody, which is against glycyl-tRNA synthetase [7], is generally less common than anti-Jo-1 antibody, it has a higher prevalence than anti-Jo-1 antibody in some case series of patients with ILD positive of anti-ARS antibodies (ARS-ILD) [8, 9]. Meanwhile, over 90% of patients with positive-anti-ARS antibodies show ILD complications [10].

On computed tomography (CT), patients with ARS-ILD often have radiologically traction bronchiectasis, consolidation and volume loss predominantly in the lower lung zones [9, 11, 12]. Histopathological patterns are reported as nonspecific interstitial pneumonia (NSIP), usual interstitial pneumonia (UIP), acute lung injury (ALI) or diffuse alveolar damage (DAD) [9, 11–13]. Although clinical course of treatment response to ARS-ILD is reportedly good, relapses are frequent [14].

However, there are only a few reports describing the clinical features of patients with ILD with positive-anti-EJ antibody (EJ-ILD) [15, 16]. The aim of this retrospective study was to define the clinical, radiological and pathological features of patients with EJ-ILD with long-term follow-up.

## Methods

### Study population

We retrospectively analyzed the medical records of 12 consecutive patients with EJ-ILD who underwent surgical lung biopsy at Kanagawa Cardiovascular and Respiratory Center between March 2005 and April 2013 and were subsequently followed up for over one year. Anti-ARS antibodies were detected with an immunoprecipitation assay at Tokai University School of Medicine as previously described method [17].

### Clinical information

We obtained the clinical presentations, physical findings, laboratory findings and pulmonary function tests at the initial visit from patients’ medical records. The diagnosis of PM/DM was based on Bohan and Peter’s criteria [18]. Patients satisfying at least two of the five of the criteria were diagnosed with PM/DM. IPAF and anti-synthetase syndrome were diagnosed according to each proposed criteria [2, 19]. The onset of EJ-ILD was divided into two types according to the time from the initial symptoms to the initial hospital visit: 1) within three months (acute onset) and 2) over three months (chronic onset). The pattern of ILD onset in PM/DM patients was divided into three types: 1) PM/DM was diagnosed at least three months earlier than ILD (PM/DM-preceding type), 2) ILD was diagnosed at least three months earlier than PM/DM (ILD-preceding type), and 3) both occurred within three months (simultaneous type) [14].

### Radiological analysis

CT images before biopsy were obtained from all the 12 patients. All images were reviewed independently by two experienced radiologists without any prior knowledge of the clinical and pathological information.

To determine the distribution and extent of parenchymal abnormalities, each lung was divided into three zones of upper, middle and lower at the level of the carina and the left inferior pulmonary vein. The following high resolution CT (HRCT) findings were coded as present or absent in each zone: reticulation, ground-glass opacity, consolidation, bronchovascular thickening, traction bronchiectasis, honeycombing, volume loss and emphysema. Radiological assessment was based on those from previous studies [9, 20]. Disagreements between the two radiologists were resolved by consensus.

### Pathological analysis

Biopsy was performed in all patients. Multiple specimens mainly from the upper and lower lobes were obtained from 11 of 12 patients. All the specimens were stained with hematoxylin-eosin and Elastica van Gieson and were reviewed independently by two experienced lung pathologists who were not aware of the clinical and radiological findings. Pathological patterns were diagnosed according to the 2002 and 2013 ATS/ERS consensus classification of the idiopathic interstitial pneumonias and 2008 ATS project for idiopathic NSIP [21, 22].

The intensity and extent of the following pathological features were semi-quantitatively graded as absent, mild, moderate or severe: pleural fibrosis, pleuritis, lymphoid follicles with germinal center, organizing pneumonia, alveolar epithelial injury, interstitial cellular infiltration, alveolar wall fibrosis, microscopic honeycombing, fibroblastic focus and collapse. Pathological assessment was based on those from previous studies [13, 23, 24].

Disagreements between the two pathologists were discussed until consensus was reached.

### Clinical course and treatment response

The clinical course and treatment response of EJ-ILD was evaluated on the basis of the degree of changes in both chest images and pulmonary functions. Two radiologists evaluated the chest radiograph and/or HRCT. We graded the changes in pulmonary functions according to the 2000 ATS/ERS consensus statement for idiopathic pulmonary fibrosis (Table 1) [25]. In addition, the pattern of disease behavior was divided into the following five types: 1) reversible and selflimited, 2) reversible disease with risk of progression, 3) stable with residual disease, 4) progressive, irreversible disease with potential for stabilization, and 5) progressive, irreversible disease despite therapy [22].

**Table 1:** Definition of the initial response to treatment

		Evaluation of pulmonary function test^a^		
		Improved	Stable	Deteriorated
Evaluation of images	Improved	Improved	Improved	Deteriorated
	Stable	Improved	Stable	Deteriorated
	Deteriorated	Improved	Deteriorated	Deteriorated

^a^“Improved” meant at least 10% increase in forced vital capacity % predicted (%FVC) or at least 15% increase in diffusing capacity of the lung for carbon monoxide % predicted (%DL_CO_). Stable was defined as within 10% change of %FVC and within 15% change of %DL_CO_. “Deteriorated” meant over 10% decrease in %FVC or over 15% decrease in %DL_CO_.

The initial response was assessed within six months after the initiation of treatment. After the initial evaluation, we assessed all chest radiographs and pulmonary functions until the end of the follow-up period. The relapse of ILD meant that the patients with deterioration needed to reinforced therapy for ILD.

## Results

### Clinical and laboratory findings of EJ-ILD

The summary of the clinical characteristics of the 12 patients is listed in Table 2. The patients included five males and seven females with median age of 62 years (range, 47–75 years) at the initial visit. Eight patients presented acute onset.

**Table 2:** Clinical characteristics and diagnosis of 12 patients

Clinical characteristics	*n* = 12
Male/female	5/7
Age at initial visit [years]	62 (47–75)
Acute onset/chronic onset	8/4
Smoking status (current or former/never smoker)	6/6
Serological findings (reference value)	*n* = 12
CK [U/L] (62–287)	140 (17–672)
LDH [U/L] (70–139)	252 (212–337)
ESR [mm/h] (2–10)	38 (17–116)
CRP [mg/dl] (0–0.1)	0.63 (0.03–1.79)
KL-6 [U/ml] (0–500)	991 (514–3208)
SP-D [ng/ml] (0–110)	232.3 (56.1–723.1)
PaO_2_ [Torr] (74–108)	71.5 (53.0–84.5)
Pulmonary functions	*n* = 11
FVC % predicted [%]	71.5 (42.4–86.1)
DL_CO_ % predicted [%]	66.1 (56.0–112.2)

Data are presented as n, or median (range)

*CK*: Creatine kinase, *LDH*: Lactate dehydrogenase, *ESR*: Erythrocyte sedimentation rate, *CRP*: C-reactive protein, *KL-6*: Klebs von den lungen-6, *SP-D*: Surfactant protein D, *PaO*_*2*_: Arterial oxygen pressure, *FVC*: Forced vital capacity, *DL*_*CO*_: Diffusion capacity for carbon monoxide

At the disease onset, extra-pulmonary symptoms were observed in only five patients, whereas all patients presented pulmonary symptoms. Only one patient was suffering from muscle weakness. Among six patients diagnosed with IPAF and anti-synthetase syndrome, no patient had skin involvement, and only one patient presented articular symptoms. Two patients have not had extra-pulmonary symptoms during entire follow-up period (Table 3). Palmar telangiectasia was not assessed in all patients.

**Table 3:** Clinical symptoms and physical findings related to IPAF and anti-synthetase syndrome

Patient number	Diagnosis	Cough	Dyspnea	Mechanic’s hand, Gottron’s sign	Heliotrope rash	Raynaud’s phenomenon	Articular involvement^a^	Unexplained digital oedema	Unexplained fever	Muscular involvement^b^	Distal digital tip ulceration	CK level
1	IPAF/ASS	+	-	-	-	-	-	-	-	-	-	218
2	IPAF/ASS	+	+	-	-	-	+	-	-	-	-	136
3	IPAF/ASS	+	-	-	-	-	-	-	-	-	-	50
4	PM^I^	+	-	-	-	-	+^c^	-	-	+^c^	-	346^E^
5	IPAF/ASS	+	+	-	-	-	-	-	+^c^	+^c^	-	51
6	DM^I^	+	-	+	-	+^c^	-	-	+^c^	-	-	143
7	IPAF/ASS	+	+	-	-	-	-	-	+^c^	+^c^	-	17
8	PM^I^	+	-	+^c^	-	+^c^	+^c^	+^c^	-	-	-	513^E^
9	DM^I^	-	+	+^c^	-	+^c^	+^c^	-	-	+^c^	-	137
10	IPAF/ASS	+	+	-	-	-	-	-	+	+^c^	-	22
11	PM^I^	+	+	-	-	-	+	+	-	+^c^	-	230
12	PM^S^	+	+	-	-	-	-	-	+	+	-	672^E^

^a^inflammatory arthritis, or polyarticular morning joint stiffness > 60mins, ^b^proximal muscular weakness, or myalgia, *CK*: creatine kinase, *IPAF*: interstitial pneumonia with autoimmune features, *ASS*: anti-synthetase syndrome, *PM*: polymyositis, *DM*: dermatomyositis, *I*: interstitial lung disease preceded PM/DM, *S*: simultaneous onset of PM and interstitial lung disease, ^c^symptoms occurred during follow up, *E*: The CK level is greater than reference value

Creatine kinase levels were elevated in three patients and lactate dehydrogenase levels were elevated in eight, while all patients presented with the elevation of erythrocyte sedimentation rate, C-reactive protein, Klebs von den Lungen-6 and surfactant protein-D levels. Electromyography, muscle biopsy and magnetic resonance imagings of muscle were performed in only two DM patients.

Specific autoantibodies other than anti-EJ antibody were found in only one patient who had anti-SSA antibody at the initial visit and was diagnosed with Sjögren’s syndrome 20 months after the diagnosis of EJ-ILD. Other patients had neither other autoantibodies nor the diagnosis of any other collagen vascular diseases except for PM/DM.

Six patients were diagnosed with PM/DM during the disease course and five of them were of the ILD-preceding type. Others were all diagnosed with idiopathic interstitial pneumonia and satisfied with the proposed criteria of both IPAF and anti-synthetase syndrome.

### Radiological findings

**Fig. 1:**
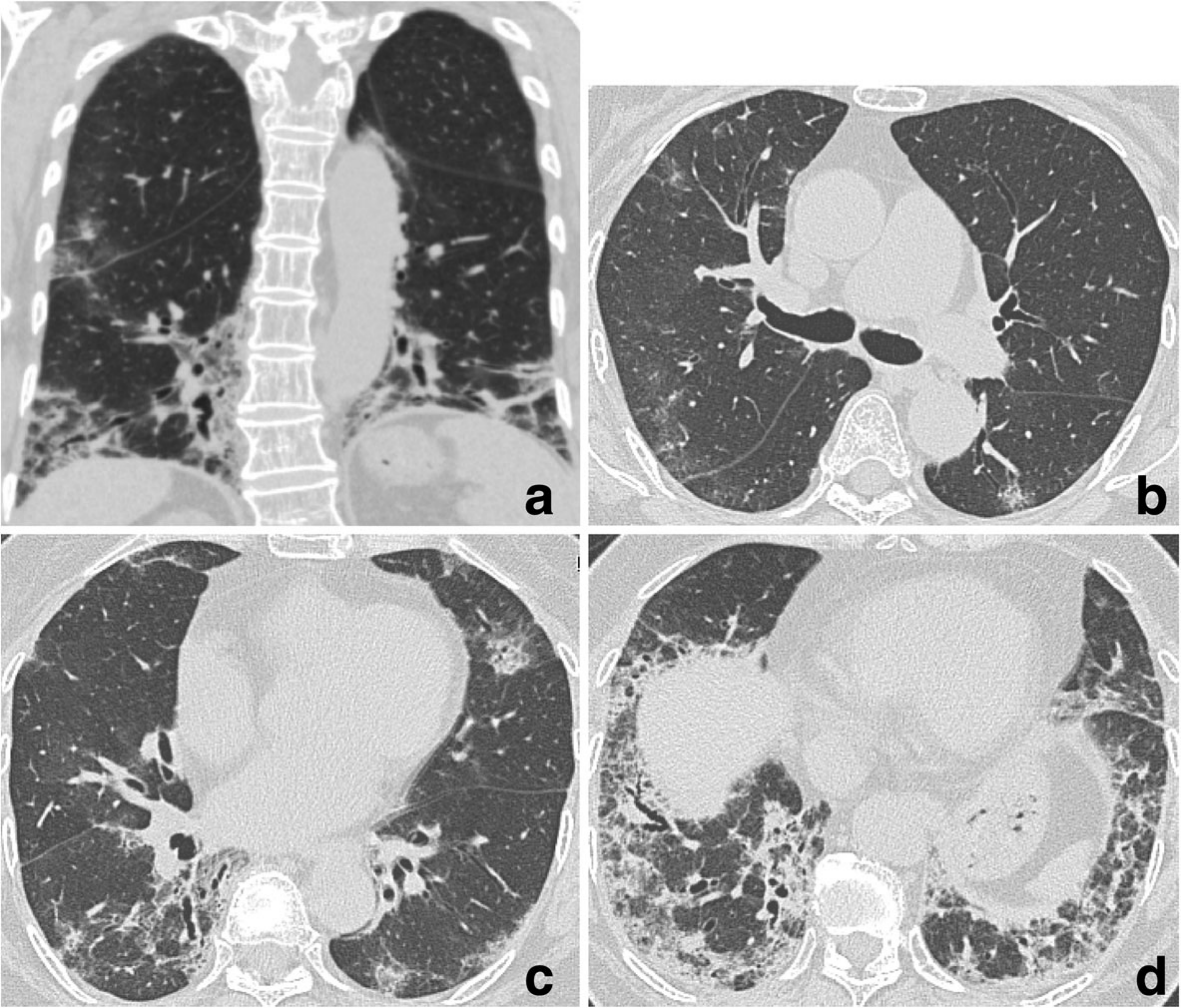
HRCT images of patient #9 (76-years-old female, pathologically cellular NSIP). **a** The coronal image of the high-resolution computed tomography (HRCT) showed bilateral volume loss and infiltration of lower lung field. **b**-**d** Transverse images of the HRCT. Consolidation was observed from middle area to the subpleural area. Traction bronchiectasis was noted inside of consolidation. Reticulation and ground-glass opacity were distributed around consolidation

The HRCT findings are described in Table 4. All findings were observed in both lobes. All but one patient presented with reticulation and ground-glass opacity in all lobes. On the other hand, volume loss, bronchovascular thickening, traction bronchiectasis and consolidation were not common in the upper zone, but were predominant in the middle or lower zones in almost all patients. Consolidation was observed along the bronchus from the middle to the subpleural area. Bronchiectasis was noted inside the consolidation, which encompassed the lung architecture. Reticulation and ground-glass opacity were distributed around the consolidation. Typical HRCT images are shown in Fig. 1.

**Table 4:** HRCT findings of 12 patients

Findings	Zones		
	Upper	Middle	Lower
Reticulation	11	12	12
Ground-glass opacity	11	12	12
Volume loss	2	9	12
Bronchovascular thickening	1	10	12
Traction bronchiectasis	5	11	11
Consolidation	4	11	11
Honeycombing	1	1	4
Emphysema	4	1	1

Upper: between apex and carina, Middle: between carina and left inferior pulmonary vein, Lower: between inferior pulmonary vein and diaphragm

### Pathological findings

**Fig. 2:**
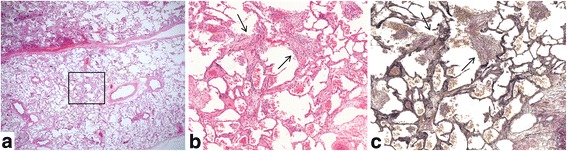
Histopathology of surgical lung biopsies of patient #10 (cellular NSIP, 62-years-old female, right S9). **a** Low power view of the specimen showed panlobular and homogeneous pattern including interstitial cellular infiltration and organization located in airspace or alveolar septa. **b**, **c** High power view of an area of a square of (**a**) revealed focal membranous organization (*arrows*) observed in the alveolar duct. Ill-defined border between the alveolar septa and alveolar lumina was seen. Staining: (**a**, **b**) Hematoxylin and eosin stain, (**c**) Elastica van Gieson stein, Magnification: (**a**) 1×, (**b**, **c**) 10×

**Fig. 3:**
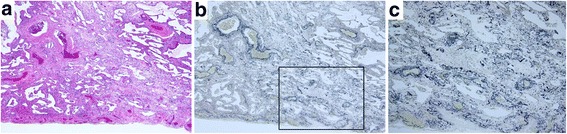
Histopathology of surgical lung biopsies of patient #6 (fibrosing NSIP, 72-years-old female, left S9). **a**, **b** Uniform fibrosis of the same age was observed diffusely in alveolar septa. Collapse was observed partially (*square*). **c** A higher magnification of an area of a square of (**b**) showing intraluminal and mural incorporation fibrosis and condensed elastic fiber of the alveolar walls. Staining: (**a**) Hematoxylin and eosin stain, (**b**, **c**) Elastica van Gieson stein, Magnification: (**a**, **b**) 4×, (**c**) 10×

**Fig. 4:**
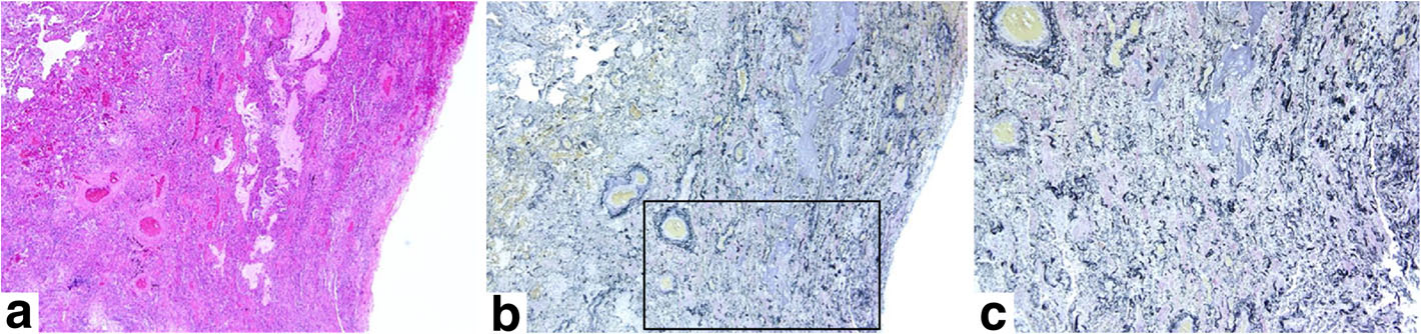
Histopathology of surgical lung biopsies of patient #3 (unclassifiable interstitial pneumonia, 60-years-old male, right S2). The specimen showed mixed fibrosing NSIP pattern and marked collapse with slight inflammatory cell infiltration. **a**, **b** Collapse with obliterative intraalveolar fibrosis and disrupted alveolar elastic fibers were observed in the *square*. **c** Fragmented elastic fibers of the alveolar walls were seen in a higher magnification of an area of a *square* of (**b**). Staining: (**a**) Hematoxylin and eosin stain, (**b**, **c**) Elastica van Gieson stein, Magnification: (**a**, **b**) 4×, (**c**) 10×

The median interval between the date of HRCT and biopsy was 28 days (range, 1–85 days). Four patients were pathologically diagnosed with cellular NSIP (c-NSIP), five were with fibrosing NSIP (f-NSIP), and three were with unclassifiable interstitial pneumonia. Organizing pneumonia, alveolar epithelial injury, interstitial cellular infiltration, pleural fibrosis and collapse with moderate or severe grade were observed in more than half of the patients (Table 5). Especially organizing pneumonia and alveolar epithelial injury with moderate or severe grade were observed in two of four patients with c-NSIP and in four of five f-NSIP patients. Collapse was observed with moderate grade in one of four patients with c-NSIP and with moderate or severe grade in four of five with f-NSIP. Acute inflammatory findings such as organizing pneumonia, alveolar epithelial injury and interstitial cellular infiltration surrounded by collapse with few hyaline membrane were more prominent in unclassifiable interstitial pneumonia than in c-NSIP or f-NSIP. Organizing pneumonia, alveolar epithelial injury and collapse did not show diffuse but rather focal distribution. On the other hand, interstitial cellular infiltration and pleural fibrosis were diffuse. Typical pathological images are shown in Figs. 2, 3, 4.

**Table 5:** Pathological diagnosis and findings

Diagnosis	*n* = 12			
cellular-NSIP	4			
fibrosing-NSIP	5			
unclassifiable interstitial pneumonia	3			
Findings	absent	mild	moderate	severe
Organizing pneumonia	1	0	7	4
Alveolar epithelial injury	1	1	8	2
Interstitial cellular infiltration	0	4	5	3
Pleural fibrosis	0	4	8	0
Collapse	1	4	6	1
Alveolar wall fibrosis	3	4	4	1
Pleuritis	2	9	1	0
Lymphoid follicles with germinal centers	6	6	0	0
Fibroblastic focus	8	2	2	0
Microscopic honeycombing	10	2	0	0

Organizing pneumonia involved less than 20% of the overall biopsy specimen when the diagnosis was NSIP [21]. Alveolar epithelial injury was defined as obscured border between alveolar septum and alveolar lumina with alveolar epithelial shedding, intra-alveolar cellular infiltration and membranous organization of the alveolar ducts and alveolar sac which meant focal acute lung injury. Collapse meant atelectatic alveoli with intra-alveolar fibrosis and agglutination of alveoli [35].

*NSIP*: nonspecific interstitial pneumonia

Organizing pneumonia and alveolar epithelial injury with moderate or severe grade were observed not only in patients with acute onset but also in those with chronic onset (Table 6).

**Table 6:** Number of patients of each pathological finding with moderate or severe grade

Findings	acute onset (*n* = 8)	chronic onset (*n* = 4)
Organizing pneumonia	8	3
Alveolar epithelial injury	6	4
Interstitial cellular infiltration	6	2
Pleural fibrosis	5	3
Collapse	3	4
Alveolar wall fibrosis	2	3
Pleuritis	0	1
Lymphoid follicles with germinal centers	0	0
Fibroblastic focus	0	2
Microscopic honeycombing	0	0

### Clinical course and treatment response

Details on the clinical courses and therapy of the 12 patients are shown in Table 7. Seven patients were treated with combination therapy of corticosteroid and immunosuppressant as the initial treatment while five were given corticosteroid monotherapy. The initial dose of corticosteroid was 0.5 mg/kg/day of prednisone in 11 patients. The median time of prednisone dose tapering to 10 mg/day was 91 days (range, 60–132 days).

**Table 7:** The clinical courses and therapy of the 12 patients

Patient number	Response to initial therapy	Disease onset	Diagnosis	Pathological diagnosis	Initial therapy	Observation period (months)	Period from initial therapy to relapse (months)	Therapy at relapse^a^	Prognosis	Disease behavior
1	improved	acute	IIP	c-NSIP	PDN	29	-	-	alive	A
2	improved	acute	IIP	unclassifiable IP	PDN + CyA	27	-	-	alive	A
3	improved	acute	IIP	unclassifiable IP	PDN + TAC	90	-	-	alive	A
4	improved	chronic	PM	f-NSIP	Low-PDN	72	-	-	alive	A
5	improved	chronic	IIP	c-NSIP	PDN + TAC	76	-	-	alive	A
6^SS^	improved	chronic	DM	f-NSIP	PDN + TAC	19	-	-	alive	A
7	relapsed	acute	IIP	f-NSIP	PDN	94	29	PDN	alive	B
8	relapsed	acute	PM	f-NSIP	PDN^c^	82	61	PDN	alive	B
9	relapsed	acute	DM	c-NSIP	PDN^c^	70	8	PDN	alive	B
10	relapsed	acute	IIP	c-NSIP	PDN^c^ + CyA	115	46	PDN + CyA	alive	B
11	relapsed	chronic	PM	f-NSIP	PDN + AZA	104	78	PDN	alive	B
12	deteriorated	acute	PM^b^	unclassifiable IP	PDN + CyA	17	-	-	dead	C

^a﻿^Patients have received these therapies until ILD relapsed., *SS*: Sjögren’s syndrome, *IIP*: idiopathic interstitial pneumonia, *PM*: polymyositis, *DM*: dermatomyositis, ^b^simultaneous onset of PM and interstitial lung disease, *c-NSIP*: cellular nonspecific interstitial pneumonia, *f-NSIP*: fibrosing nonspecific interstitial pneumonia, *IP*: interstitial pneumonia, *PDN*: 0.5 mg/kg/day of prednisone, *CyA*: cyclosporine A, *TAC*: tacrolimus, *Low-PDN*: 0.2 mg/kg/day of prednisone, ^c﻿^started with methylprednisone pulse therapy (500 mg/body), *AZA*: azathioprine, A: progressive, irreversible disease with potential for stabilization, B: reversible disease with risk of progression, C: progressive, irreversible disease despite therapy

**Fig. 5:**
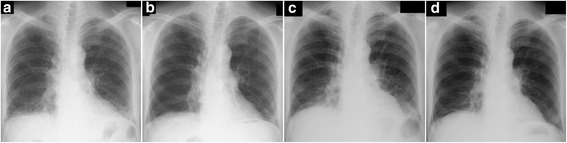
Changes of chest radiographs of patient #11. **a** The initial chest radiograph before treatment showed infiltration and volume loss of lower lung field. **b** After four months from the initiation of treatment, consolidation and volume loss improved. **c** However they arose again at the time of relapse. **d** At the end of follow-up, treatment improved infiltration and volume

**Fig. 6:**
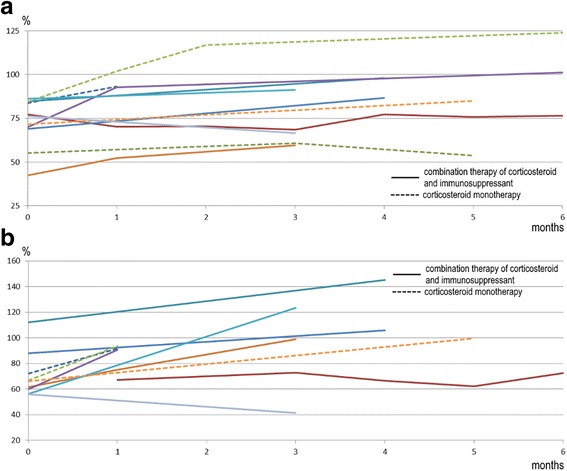
Changes of pulmonary functions of 10 patients during initial evaluation period. Pulmonary function improved or was stable in nine patients. **a** Line graph of forced vital capacity % predicted. **b** Line graph of diffusing capacity of the lung for carbon monoxide % predicted

The initial response was evaluated as improved in 11 of 12 patients. Radiological findings improved in 11 of 12 patients. Representative images of chest radiograph are shown in Fig. 5, indicating the apparent lung volume recovery. Pulmonary function, evaluated in 10 patients, improved or stabilized in nine patients. Forced vital capacity % predicted (%FVC) and/or diffusing capacity of the lung for carbon monoxide % predicted (%DL_CO_) increased immediately after the initial treatment and remained almost stable between the initial evaluation period (Fig. 6). On disease behavior, the type of reversible disease with risk of progression were six patients, progressive and irreversible disease with potential for stabilization were five patients, and progressive and irreversible disease despite therapy were one patient. Patient #12 was the only patient who did not respond to the treatment and died during the follow-up period.

**Fig. 7:**
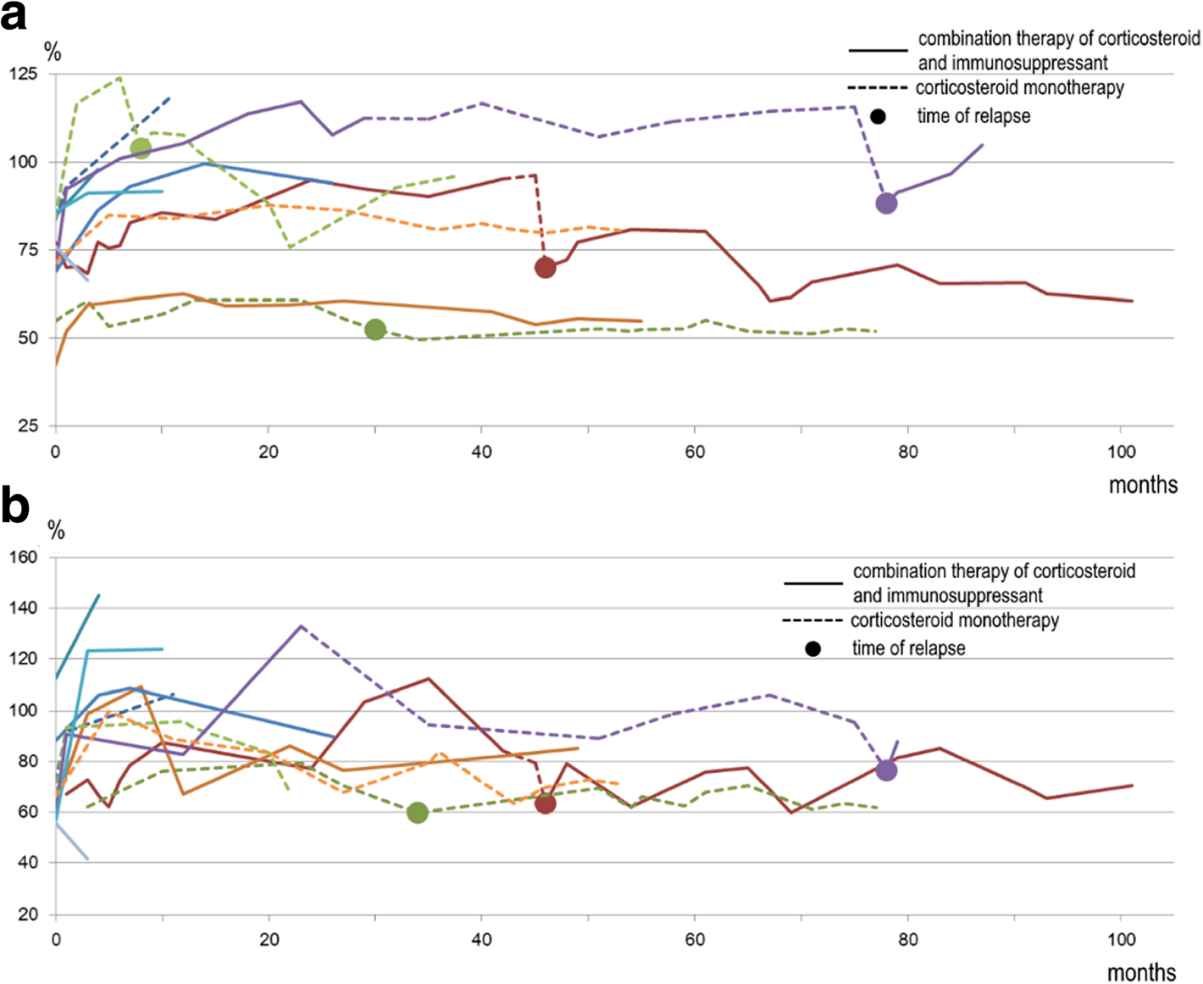
Changes of pulmonary functions of 10 patients during whole follow up period. Four patients deteriorated with corticosteroid monotherapy. **a** Line graph of forced vital capacity % predicted. **b** Line graph of diffusing capacity of the lung for carbon monoxide % predicted

The median follow-up time was 74 months (range, 17–115 months). ILD relapsed in five of the 11 improved patients after a median of 46 months (range, 8–78 months) from treatment initiation. Among the five relapsed patients, four were on prednisone monotherapy and four presented with acute onset of the disease. There were few differences in the changes on chest radiograph and pulmonary function test between the relapsed patients and non-relapsed patients until relapse. However, upon relapse, the loss of lung volume was clearly recognized in chest radiograph, and both %FVC and %DL_CO_ significantly decreased. The addition of immunosuppressant and/or the increase of the dose of corticosteroid reduced the abnormal shadow and prevented deterioration of %FVC and %DL_CO_ (Fig. 7). On the other hand, four of the six patients without relapse had continuously been on combination therapy of corticosteroid and immunosuppressant.

## Discussion

We retrospectively evaluated the clinical, radiological, pathological features and long-term clinical courses of 12 patients with EJ-ILD who underwent surgical lung biopsy. We have shown that patients with EJ-ILD had often acute onset of ILD with lower-lung predominant shadows with volume loss on HRCT, pathologically NSIP or unclassifiable interstitial pneumonia with acute inflammatory findings, and good response for initial steroid therapy, although relapse was frequent among the patients with corticosteroid monotherapy. In most patients with EJ-ILD clinical disease behavior was reversible or progressive and irreversible disease. Although anti-EJ antibodies correlated well with ILD, there are a few reports which describe the clinical, radiological and pathological findings of EJ-ILD and no reports describe any detailed clinical courses [16, 26]. To our knowledge, this is the first study to describe the detailed clinical features of biopsy-proven EJ-ILD with long-term follow-up period.

The onset of EJ-ILD was often acute in the present study. EJ-ILD progressed within three months in previous literature [15, 16]. Fifty to 70% of patients with anti-ARS antibodies other than anti-EJ antibody reportedly presented with chronic onset of ILD [12, 13, 27–29]. This suggests that onset of the disease varies among ARS-ILD, depending on the type of specific antibody.

Fifty percent of patients in this cohort were ultimately diagnosed with PM/DM, which was consistent with the previous report [8, 10]. However, our study showed that five of six PM/DM patients had ILD-preceding type, whereas only 30%–35% of patients with anti-ARS antibodies were reportedly ILD-preceding disease [12, 14, 17, 28].

Regarding to IPAF and anti-synthetase syndrome, almost no patient presented physical symptoms at initial visit. However, a half of them developed muscular symptoms and fever during follow-up period. This result is also compatible with the previous report [10]. These suggest that it is difficult to distinguish anti-synthetase syndrome from IPAF at initial visit and pulmonary symptoms precede in some patients with anti-EJ antibody before developing anti-synthetase syndrome or PM/DM.

The HRCT findings were basically compatible with those of previous reports on ARS-ILD and ILD associated with PM/DM [9, 20, 30]. Our study revealed consolidation and volume loss along the bronchovascular bundle predominantly in the middle and lower lung zones. These findings were similar to the radiological NSIP pattern [21]. In some cases, changes in lung volume on chest radiograph correlated well with lung function and disease condition. Chest radiograph may be an adjunct indicator when chest CT is unavailable.

Pathologically unclassifiable interstitial pneumonia and NSIP were observed in our study although recent reports showed that the histopathological patterns of ARS-ILD were NSIP, UIP, ALI and DAD [11, 13, 15, 16]. Acute inflammatory findings have not been shown in ARS-ILD patients with chronic onset in previous literature [13, 15, 16]. We here showed that acute inflammatory findings focally distributed with few hyaline membrane were observed not only in acute onset but also in chronic onset. In this point, our results support the recent reports. The pathological diagnosis of EJ-ILD may not match but resemble ALI and DAD because these were reported as indicating the diffuse and uniform widespread involvement of the pulmonary parenchyma and the pulmonary lobule with hyaline membrane [15, 16, 31, 32]. Moreover, the high prevalence of acute inflammatory findings and ILD distribution seem to suggest very similar type ILD as in other anti-synthetase antibodies. However, we cannot reliably draw a conclusion because of a small cohort size.

In this study, the initial treatment response of EJ-ILD and the rate of relapse were similar to those previously reported for ARS-ILD [14]. Although the initial treatment for EJ-ILD was effective for most of the patients, ILD relapsed in nearly half of them. These findings suggest that the patients with EJ-ILD may have the potential high risk of disease progression. We found that those with relapse presented with NSIP, often on acute onset and were on corticosteroid monotherapy in this cohort, whereas the rate of relapse of ILD was lower in patients on combination therapy. To date, it has also been shown that in ARS-ILD the corticosteroid dose reduction might relate to the relapse [12, 14], and combination therapy of corticosteroid and tacrolimus was effective [33]. The combination therapy was also recommended to treat ILD related to PM/DM [34]. These suggest that combination therapy of corticosteroid and immunosuppressant may be preferable to corticosteroid monotherapy especially in case of acute-onset ILD.

This study has several limitations. First, it was a retrospective study with a small sample size. Second, since this study was performed in a specialized single-center for the respiratory diseases, there might be a selection bias for the diagnosis of PM/DM at the initial visit. However, in fact, all patients except patient #12 had muscle weakness at the initial visit.

## Conclusions

We delineated the long-term clinical characteristics of EJ-ILD. Because of the diversity of the clinical course, combination therapy of corticosteroid and immunosuppressant should be argued on a case-by-case basis to prevent relapse of EJ-ILD. We need to conduct long-term observation to assess clinical behavior and prevent complications of EJ-ILD.

## Abbreviations

%DL_CO_: Diffusing capacity of the lung for carbon monoxide % predicted
%FVC: Forced vital capacity % predicted
ALI: Acute lung injury
ARS: Aminoacyl-tRNA synthetase
ARS-ILD: Interstitial lung disease with positive-anti-ARS antibodies
ATS: American Thoracic Society
c-NSIP: Cellular nonspecific interstitial pneumonia
CT: Computed tomography
DAD: Diffuse alveolar damage
EJ-ILD: Interstitial lung disease with positive-anti-EJ antibodies
ERS: European Respiratory Society
f-NSIP: Fibrosing nonspecific interstitial pneumonia
HRCT: High-resolution computed tomography
ILD: Interstitial lung disease
IPAF: Interstitial pneumonia with autoimmune features
NSIP: Nonspecific interstitial pneumonia
PM/DM: Polymyositis/dermatomyositis
UIP: Usual interstitial pneumonia

## References

[1] Kinder BW, Collard HR, Koth L, Daikh DI, Wolters PJ, Elicker B, Jones KD, King TE (2007). Idiopathic nonspecific interstitial pneumonia: lung manifestation of undifferentiated connective tissue disease?. Am J Respir Crit Care Med.

[2] Fischer A, Antoniou KM, Brown KK, Cadranel J, Corte TJ, du Bois RM, Lee JS, Leslie KO, Lynch DA, Matteson EL (2015). An official European Respiratory Society/American Thoracic Society research statement: interstitial pneumonia with autoimmune features. Eur Respir J.

[3] Lega JC, Fabien N, Reynaud Q, Durieu I, Durupt S, Dutertre M, Cordier JF, Cottin V (2014). The clinical phenotype associated with myositis-specific and associated autoantibodies: a meta-analysis revisiting the so-called antisynthetase syndrome. Autoimmun Rev.

[4] Mimori T, Imura Y, Nakashima R, Yoshifuji H (2007). Autoantibodies in idiopathic inflammatory myopathy: an update on clinical and pathophysiological significance. Curr Opin Rheumatol.

[5] Targoff IN (1994). Immune manifestations of inflammatory muscle disease. Rheum Dis Clin North Am.

[6] Hirakata M, Nagai S (2000). Interstitial lung disease in polymyositis and dermatomyositis. Curr Opin Rheumatol.

[7] Targoff IN, Trieu EP, Plotz PH, Miller FW (1992). Antibodies to glycyl-transfer RNA synthetase in patients with myositis and interstitial lung disease. Arthritis Rheum.

[8] Takato H, Waseda Y, Watanabe S, Inuzuka K, Katayama N, Ichikawa Y, Yasui M, Fujimura M (2014). Pulmonary manifestations of anti-ARS antibody positive interstitial pneumonia--with or without PM/DM. Respir Med.

[9] Watanabe K, Handa T, Tanizawa K, Hosono Y, Taguchi Y, Noma S, Kobashi Y, Kubo T, Aihara K, Chin K (2011). Detection of antisynthetase syndrome in patients with idiopathic interstitial pneumonias. Respir Med.

[10] Hamaguchi Y, Fujimoto M, Matsushita T, Kaji K, Komura K, Hasegawa M, Kodera M, Muroi E, Fujikawa K, Seishima M (2013). Common and distinct clinical features in adult patients with anti-aminoacyl-tRNA synthetase antibodies: heterogeneity within the syndrome. PLoS One.

[11] Fischer A, Swigris JJ, du Bois RM, Lynch DA, Downey GP, Cosgrove GP, Frankel SK, Fernandez-Perez ER, Gillis JZ, Brown KK (2009). Anti-synthetase syndrome in ANA and anti-Jo-1 negative patients presenting with idiopathic interstitial pneumonia. Respir Med.

[12] Koreeda Y, Higashimoto I, Yamamoto M, Takahashi M, Kaji K, Fujimoto M, Kuwana M, Fukuda Y (2010). Clinical and pathological findings of interstitial lung disease patients with anti-aminoacyl-tRNA synthetase autoantibodies. Intern Med.

[13] Yousem SA, Gibson K, Kaminski N, Oddis CV, Ascherman DP (2010). The pulmonary histopathologic manifestations of the anti-Jo-1 tRNA synthetase syndrome. Mod Pathol.

[14] Yoshifuji H, Fujii T, Kobayashi S, Imura Y, Fujita Y, Kawabata D, Usui T, Tanaka M, Nagai S, Umehara H (2006). Anti-aminoacyl-tRNA synthetase antibodies in clinical course prediction of interstitial lung disease complicated with idiopathic inflammatory myopathies. Autoimmunity.

[15] Hara Y, Tanaka T, Tabata K, Shiraki A, Hayashi K, Kashima Y, Hayashi T, Fukuoka J (2014). Anti-glycyl tRNA synthetase antibody associated interstitial lung disease without symptoms of polymyositis/dermatomyositis. Pathol Int.

[16] Schneider F, Yousem SA, Bi D, Gibson KF, Oddis CV, Aggarwal R (2014). Pulmonary pathologic manifestations of anti-glycyl-tRNA synthetase (anti-EJ)-related inflammatory myopathy. J Clin Pathol.

[17] Sato S, Kuwana M, Hirakata M (2007). Clinical characteristics of Japanese patients with anti-OJ (anti-isoleucyl-tRNA synthetase) autoantibodies. Rheumatology (Oxford).

[18] Bohan A, Peter JB (1975). Polymyositis and dermatomyositis (second of two parts). N Engl J Med.

[19] Lega JC, Reynaud Q, Belot A, Fabien N, Durieu I, Cottin V (2015). Idiopathic inflammatory myopathies and the lung. Eur Respir Rev.

[20] Mino M, Noma S, Taguchi Y, Tomii K, Kohri Y, Oida K (1997). Pulmonary involvement in polymyositis and dermatomyositis: sequential evaluation with CT. AJR Am J Roentgenol.

[21] Travis WD, Hunninghake G, King TE, Lynch DA, Colby TV, Galvin JR, Brown KK, Chung MP, Cordier JF, du Bois RM (2008). Idiopathic nonspecific interstitial pneumonia: report of an American Thoracic Society project. Am J Respir Crit Care Med.

[22] Travis WD, Costabel U, Hansell DM, King TE, Lynch DA, Nicholson AG, Ryerson CJ, Ryu JH, Selman M, Wells AU (2013). An official American Thoracic Society/European Respiratory Society statement: Update of the international multidisciplinary classification of the idiopathic interstitial pneumonias. Am J Respir Crit Care Med.

[23] Fischer A, West SG, Swigris JJ, Brown KK, du Bois RM (2010). Connective tissue disease-associated interstitial lung disease: a call for clarification. Chest.

[24] Vij R, Noth I, Strek ME (2011). Autoimmune-featured interstitial lung disease: a distinct entity. Chest.

[25] American Thoracic Society (2000). Idiopathic pulmonary fibrosis: diagnosis and treatment. International consensus statement. American Thoracic Society (ATS), and the European Respiratory Society (ERS). Am J Respir Crit Care Med.

[26] Hara H, Inoue Y, Sato T (2005). Clinical and pathological findings of patients with interstitial lung disease associated with antisynthetase. Nihon Kokyuki Gakkai Zasshi.

[27] Hervier B, Wallaert B, Hachulla E, Adoue D, Lauque D, Audrain M, Camara B, Fournie B, Couret B, Hatron PY (2010). Clinical manifestations of anti-synthetase syndrome positive for anti-alanyl-tRNA synthetase (anti-PL12) antibodies: a retrospective study of 17 cases. Rheumatology (Oxford).

[28] Marie I, Hachulla E, Cherin P, Dominique S, Hatron PY, Hellot MF, Devulder B, Herson S, Levesque H, Courtois H (2002). Interstitial lung disease in polymyositis and dermatomyositis. Arthritis Rheum.

[29] Tillie-Leblond I, Wislez M, Valeyre D, Crestani B, Rabbat A, Israel-Biet D, Humbert M, Couderc LJ, Wallaert B, Cadranel J (2008). Interstitial lung disease and anti-Jo-1 antibodies: difference between acute and gradual onset. Thorax.

[30] Debray MP, Borie R, Revel MP, Naccache JM, Khalil A, Toper C, Israel-Biet D, Estellat C, Brillet PY (2015). Interstitial lung disease in anti-synthetase syndrome: initial and follow-up CT findings. Eur J Radiol.

[31] Katzenstein AL, Bloor CM, Leibow AA (1976). Diffuse alveolar damage--the role of oxygen, shock, and related factors. A review Am J Pathol.

[32] Katzenstein AL, Myers JL, Mazur MT (1986). Acute interstitial pneumonia. A clinicopathologic, ultrastructural, and cell kinetic study. Am J Surg Pathol.

[33] Wilkes MR, Sereika SM, Fertig N, Lucas MR, Oddis CV (2005). Treatment of antisynthetase-associated interstitial lung disease with tacrolimus. Arthritis Rheum.

[34] Takada K, Kishi J, Miyasaka N (2007). Step-up versus primary intensive approach to the treatment of interstitial pneumonia associated with dermatomyositis/polymyositis: a retrospective study. Mod Rheumatol.

[35] Burkhardt A (1989). Alveolitis and collapse in the pathogenesis of pulmonary fibrosis. Am Rev Respir Dis.

